# Discovery and Biochemical Characterization of PlyP56, PlyN74, and PlyTB40—*Bacillus* Specific Endolysins

**DOI:** 10.3390/v10050276

**Published:** 2018-05-21

**Authors:** Irina Etobayeva, Sara B. Linden, Farhang Alem, Laith Harb, Lucas Rizkalla, Philip D. Mosier, Allison A. Johnson, Louise Temple, Ramin M. Hakami, Daniel C. Nelson

**Affiliations:** 1 Institute for Bioscience and Biotechnology Research, Rockville, MD 20850, USA; romani@ibbr.umd.edu (I.E.); slinden1@umd.edu (S.B.L.); 2 Department of Veterinary Medicine, University of Maryland, College Park, MD 20742, USA; 3 School of Systems Biology and National Center for Biodefense & Infectious Diseases, Biomedical Research Laboratory, George Mason University, Manassas, VA 22030, USA; falem@masonlive.gmu.edu (F.A.); rhakami@gmu.edu (R.M.H.); 4 Department of Integrated Science & Technology, James Madison University, Harrisonburg, VA 22807, USA; harbla@tamu.edu (L.H.); templelm@jmu.edu (L.T.); 5 Center for the Study of Biological Complexity, Virginia Commonwealth University, Richmond, VA 23220, USA; rizkallalj@mymail.vcu.edu (L.R.); pdmosier@vcu.edu (P.D.M.); aajohnson@vcu.edu (A.A.J.); 6 Department of Medicinal Chemistry and Institute for Structural Biology, Drug Discovery and Development, School of Pharmacy, Virginia Commonwealth University, Richmond, VA 23220, USA

**Keywords:** endolysin, bacteriophage, *Bacillus cereus sensu lato*, peptidoglycan hydrolase

## Abstract

Three *Bacillus* bacteriophage-derived endolysins, designated PlyP56, PlyN74, and PlyTB40, were identified, cloned, purified, and characterized for their antimicrobial properties. Sequence alignment reveals these endolysins have an N-terminal enzymatically active domain (EAD) linked to a C-terminal cell wall binding domain (CBD). PlyP56 has a Peptidase_M15_4/VanY superfamily EAD with a conserved metal binding motif and displays biological dependence on divalent ions for activity. In contrast, PlyN74 and PlyTB40 have T7 lysozyme-type Amidase_2 and carboxypeptidase T-type Amidase_3 EADs, respectively, which are members of the MurNAc-LAA superfamily, but are not homologs and thus do not have a shared protein fold. All three endolysins contain similar SH3-family CBDs. Although minor host range differences were noted, all three endolysins show relatively broad antimicrobial activity against members of the *Bacillus cereus sensu lato* group with the highest lytic activity against *B. cereus* ATCC 4342. Characterization studies determined the optimal lytic activity for these enzymes was at physiological pH (pH 7.0–8.0), over a broad temperature range (4–55 °C), and at low concentrations of NaCl (<50 mM). Direct comparison of lytic activity shows the PlyP56 enzyme to be twice as effective at lysing the cell wall peptidoglycan as PlyN74 or PlyTB40, suggesting PlyP56 is a good candidate for further antimicrobial development as well as bioengineering studies.

## 1. Introduction

The *Bacillus* genus consists of a diverse collection of aerobic organisms that are common residents of the soil and occasionally become opportunistic pathogens of humans. *Bacillus* species are Gram-positive, rod-shaped bacilli that also form endospores, which allow their survival under adverse environmental conditions. Once these conditions are resolved, endospores germinate into vegetative bacilli to continue their life cycle. From the soil, vegetative bacilli or endospores can be transmitted to humans or animals via contaminated water and produce. Resistant to irradiation, endospores allow the bacteria to remain dormant for long periods of time on surfaces in food-processing facilities, making it virtually impossible to eliminate pathogenic bacilli from the environment [1].

Although the majority of bacilli are relatively harmless to humans and animals [2], genetically related species of the *B. cereus sensu lato* group are capable of causing clinical disease and toxin-mediated food poisoning. Among these species, the most phenotypically related are *B. cereus*, *B. anthracis*, and *B. thuringiensis* [3]. *B. cereus* is capable of producing both emetic and diarrheal toxins. These species are opportunistic pathogens and widespread food contaminants highly resilient to pasteurization efforts [4]. In addition to causing gastrointestinal conditions, *B. cereus* species are also capable of causing ocular infections [5] and catheter-associated blood stream infections [6]. *B. anthracis* is an obligate pathogen and the etiologic agent of anthrax. While this organism generally is restricted to grazing animals, systemic anthrax has a high fatality rate in humans due to secretion of a three-protein toxin. While *B. cereus* and *B. anthracis* are known for causing disease and food poisoning in humans and animals, *B. thuringiensis* is an insect pathogen and its parasporal crystal proteins are used as an insecticide [7]. Otherwise, the three members of the *B. cereus sensu lato* group have very little differences in their genomes and often share the same plasmid-associated pathogenicity genes, which make it difficult to differentiate the species from one another [8].

A growing number of reports about multidrug resistant *B. cereus* isolates in food have been reported worldwide [9,10,11], which has prompted a search for an alternative to conventional antibiotics. Bacteriophage-encoded endolysins have been researched as one such alternative [12,13]. Endolysins are enzymes encoded by the late genes during a bacteriophage replication cycle. Once synthesized, endolysins target evolutionarily conserved covalent bonds within the bacterial peptidoglycan, lysing host bacteria from the inside to allow bacteriophage progeny release into the extracellular environment [14]. Significantly, endolysins applied extrinsically also can compromise the peptidoglycan integrity in the absence of a bacteriophage delivery system [15,16,17,18].

Typically, endolysins derived from bacteriophage that infect Gram-positive hosts consist of two domains: a conserved N-terminal enzymatically active domain (EAD) fused via a short linker sequence to a C-terminal cell wall binding domain (CBD) [19]. Based on the cleavage sites of one of the major covalent bonds within the bacterial peptidoglycan polymer, EADs are divided into five conserved classes: muramidases, glucosaminidases, endopeptidases, l-alanine amidases, and lytic transglycosylases. In contrast, CBDs are diverse in sequence and confer targeted specificity to a bacterial species or strain by binding a conserved carbohydrate moiety on the bacterial cell surface [20].

In this study, we identified and characterized three novel *B. cereus* endolysins, PlyP56, PlyN74, and PlyTB40, each with different EADs but homologous CBDs. We found that these endolysins were highly active against *B. cereus* species, with lesser activity against other *Bacillus* species. Based on characterization of its biochemical properties and specificity, PlyP56 is a more effective endolysin compared to PlyN74 and PlyTB40, but all three are amenable to further bioengineering studies.

## 2. Materials and Methods

### 2.1. Bacteriophage Sequence Analysis

Forty-six sequenced *Bacillus*-specific bacteriophage genomes contained in the Bacillus Phage Database (bacillus.phagesdb.org) and GenBank were screened for putative endolysins. Each bacteriophage open reading frame (ORF) was searched with the BLASTN, BLASTP, Pfam, and CDD databases. Six published endolysin sequences (LysB4, Ply500, PlyL, PlyPSA, LysBPS13, and phi29) were added for comparison [21,22,23,24,25,26]. Phylogenetic trees were drawn using MEGA7 [27] to determine phylogenetic position of ORFs among *Bacillus* species-specific bacteriophages using the Maximum Likelihood method based on the JTT matrix-based model [28]. The percentage of trees in which the associated taxa clustered together is shown next to the branches. Initial tree(s) for the heuristic search were obtained automatically by applying Neighbor-Join and BioNJ algorithms to a matrix of pairwise distances estimated using a JTT model, and then selecting the topology with superior log likelihood value.

Genes encoding the *B. cereus* group-specific endolysins Phrodo ORF_56 (AMW62097.1), which we call PlyP56, Nigalana ORF_74 (AMW61226.1), which we call PlyN74, and TsarBomba ORF_40 (ALA13156.1), which we call PlyTB40, were selected for expression in *Escherichia coli*.

### 2.2. Bacterial Strains and Growth Conditions

*Bacillus* strains used in this study are described in Table 2. Unless otherwise described, bacterial strains were purchased from the American Type Culture Collection (ATCC). *B. anthracis* strains (both BSL2 and BSL3) were procured from the Biodefense and Emerging Infections Research Resources (BEI Resources). All BSL-3 work was performed according to the established and CDC-approved Standard Operating Procedures/Protocols (SOPs) at the BSL3 containment facility of George Mason University. All standard safety precautions were strictly followed for conduct of the studies, including use of protective personnel equipment and other personal sterile practices specifically designed for safe conduct of BSL3 work. The containment facilities at George Mason University are registered with the CDC to allow possession, use, and transfer of select agents (including *B. anthracis*) and toxins according to specified guidelines. All *Bacillus* strains were propagated in Brain Heart Infusion (BHI) plates or BHI broth at 37 °C and shaken at 200 rpm. DH5α competent and BL21 (DE3) competent *E. coli* strains were used for cloning and protein expression. *E. coli* strains were propagated overnight at 37 °C and shaken at 220 rpm unless otherwise stated. *E. coli* strains were cultured in Luria–Bertani (LB) broth (BD Biosciences, Franklin Lakes, NJ, USA), and/or on LB plates supplemented with 100 μg/mL ampicillin. All chemicals and culture media were acquired from Sigma (St. Louis, MO, USA) unless otherwise stated.

### 2.3. Cloning of Vector Constructs

Endolysin-encoding genes, *plyP56*, *plyN74*, and *plyTB40*, were codon-optimized for expression in *E. coli* and chemically synthesized by Thermo Fisher (Waltham, MA, USA) in a pMA_T vector. The constructs with EcoRI and XbaI restriction sites and a C-terminal hexahistidine tag (6xHis tag) were subcloned into an arabinose-inducible pBAD24 expression vector, sequenced (Macrogen, Seoul, South Korea) to confirm identity, and eventually transformed into BL21 (DE3) competent *E. coli.* Ampicillin resistant colonies were expanded and once again retested by sequencing. The ApE-A plasmid editor (version number 2.0.47, University of Utah, Salt Lake City, UT, USA) was utilized for DNA sequence manipulations and analysis. Alternatively, the CBDs for each endolysin, corresponding to residues 174–259 for PlyP56, 190–275 for PlyN74, and residues 191–272 for PlyTB40, were cloned identically to the procedures described above for the full-length enzymes, with the exception that the 6xHis tag was placed on the N-terminus for the CBD constructs. Primers for these constructs are contained in Supplementary Table S1.

### 2.4. Recombinant Protein Expression

Overnight cultures of *E. coli* strain BL21 (DE3) transformed with pBAD24 vectors containing *plyP56*, *plyN74*, or *plyTB40* genes, or their corresponding CBDs, were diluted 1:100 (*v*/*v*) with sterile LB broth supplemented with ampicillin (100 μg/mL) and shaken at 220 rpm and 37 °C for approximately 3 h. Once the optical density (OD_600_) reached 0.8, protein expression was induced with l-arabinose (0.25%). *E coli* cultures were returned to the shaker which was set at 180 rpm and 18 °C for overnight protein expression (~16 h). The following morning, bacterial cells were pelleted by centrifugation at 5000 rpm for 10 min at 4 °C. The supernatant was discarded and cell pellets were subjected to protein purification.

### 2.5. Recombinant Protein Purification

The cell pellets were frozen at −80 °C for 15–20 min before sonication. Frozen pellets were thawed in lysis buffer (phosphate buffered saline supplemented with 10 mM imidazole, 1 mM phenylmethylsulfonyl fluoride (PMSF), pH 7.4) with 185 rpm shaking on an orbital shaker until the pellet dissolved completely. The resulting solution was sonicated (duty cycle 30, output control 6) for 14 min to lyse cells. After sonication, cell debris was removed by centrifugation at 12,000 rpm for 45 min at 4 °C. The supernatant containing soluble protein was filtered with a 0.45 mm filter (Whatman, Maidstone, UK) and recombinant proteins were applied to Mini Profinity^TM^ IMAC Cartridges (Bio-Rad, Hercules, CA, USA) and eluted in 10 mL fractions of 20, 50, 100, 250, and 500 mM imidazole. Proteins were analyzed by SDS-polyacrylamide gel electrophoresis (SDS-PAGE) for purity. Fractions containing homologous recombinant proteins were pooled and dialyzed overnight against PBS (pH 7.4) supplemented with 300 mM NaCl. Protein concentrations were determined by the Bradford assay following manufacturer’s instructions (Bio-Rad). Purified proteins were stored at –80 °C in PBS (pH 7.4) supplemented with 15% glycerol.

### 2.6. Turbidity Reduction Assay

Bacteriolytic activity of endolysins was measured via the turbidity reduction assay as described [19]. The assay was performed in a standard 96-well titration plate (Thermo Fisher Scientific) with an overnight bacterial culture of indicator strain, *B. cereus* ATCC 4342, for all dose range and biochemical characterization studies. For all host range studies, a 4-h culture of mid-log bacteria was used. A change in OD_600_ was measured every 15 s over the duration of the assay (20 min) on a SpectraMax 190 spectrophotometer (Molecular Devices, San Jose, CA, USA). Briefly, bacterial cells were pelleted at 5000 rpm for 10 min at 4 °C and resuspended in sterile PBS. A 100 μL volume of cell suspension was added to each well containing 100 μL of each endolysin at a predetermined concentration range such that the starting OD_600_ was equal to 1.0. Wells with a mixture of only bacteria in PBS served as a negative control and established a settling baseline that was subtracted from the experimental data. Bacteriolysis was quantified as the percentage of activity relative to the lytic activity of 100 μg/mL PlyP56 on *B. cereus* ATCC 4342, which represented 100% activity for all dose range analysis, and at 50 μg/mL of PlyP56 (100% activity), for all biochemical characterization studies. All experiments were performed in triplicate on three consecutive days.

### 2.7. Plate Lysis (Spot) Assay

In addition to the turbidity reduction assays, *B. cereus* ATCC 4342 and *B. anthracis* strains were assayed via plate lysis assay. Briefly, bacterial cells were harvested and pelleted at their mid-log phase (4-h cultures). Pellets were then washed twice in PBS, resuspended in 12 mL of 0.7% semisolid agar cooled to 50 °C, poured onto square 10-cm petri dishes, and gently tilted to cover the bottom of the dish. Endolysins were serial diluted 10-fold in PBS to make the concentrations 1 mg/mL, 0.1 mg/mL, and 0.01 mg/mL. Spots (10 μL) were made across a row for 10 μg, 1 μg, 0.1 μg endolysin, and PBS with no endolysin served as a buffer control. Plates were dried in a biosafety hood for 15–20 min and incubated face up at 37 °C for 2 h. Clearing zones were assessed at 1 h and 2 h post-spotting.

### 2.8. Characterization of PlyP56, PlyN74, and PlyTB40

The turbidity reduction assays on overnight cultures described above were used to determine the optimal lytic conditions. For dose–response studies, endolysins were serially diluted beginning with a starting concentration of 100 μg/mL. To evaluate enzymatic activity over a pH range of 3.0 to 11.0, bacterial cells were diluted in equal volumes of universal pH buffer (40 mM boric acid and 40 mM phosphoric acid (BP) buffer adjusted to the desired pH with NaOH), and were challenged against each endolysin at a final concentration of 50 μg/mL. The influence of NaCl on lytic activity of endolysins at 50 μg/mL was tested in BP buffer at pH 7.4 supplemented with increasing concentrations of NaCl (0–500 mM). Kinetic stability of endolysins was evaluated as described [26], with minor modifications. Briefly, endolysins were incubated at indicated temperatures (4 °C, 25 °C, 37 °C, 45 °C, 55 °C, or 60 °C) for 30 min, recovered on ice for 5 min, and subjected to the turbidity reduction assay at previously determined optimal conditions (pH, NaCl) for each endolysin. To evaluate the role of divalent cations in catalytic function, endolysins were dialyzed overnight at 4 °C in Tris-EDTA buffer (20 mM Tris, 20 mM NaCl, 5 mM EDTA, pH 7.4) to remove any residual metal ions. Subsequently, one half of the EDTA-treated endolysins was stored overnight at 4 °C and the second half was dialyzed overnight in Tris-buffered saline (TBS) (pH 7.4) supplemented with 6 mM CaCl_2_ or 6 mM MgCl_2_. Lysis of *B. cereus* ATCC 4342 was assayed via turbidity assay and untreated endolysins served as a control. 

### 2.9. Spectrum of Lytic Activity

The host range of the endolysins was accessed via turbidity reduction assay. Overnight cultures of all bacilli were diluted 1:100 and incubated an additional 4 h in fresh media. Cultures were then exposed to each endolysin at a concentration of 100 μg/mL in the 96-well plate and lytic activities were represented as the percentage of lysis relative to 100% activity of each endolysin against the *B. cereus* ATCC 4342 indicator strain after 20 min incubation. Alternatively, the plate lysis assay described above was used to determine the host range against several *B. anthracis* strains, where +, ++, and +++ indicates an observed clearing zone for 10 μg, 1 μg, and 0.1 μg, respectively, of each endolysin.

### 2.10. Fluorescent Labeling of CBDs

Purified CBDs were chemically crosslinked to an amine-reactive AlexaFluor^®^ 555 fluorescent dye (Thermo Fisher Scientific) according to the manufacturer’s instructions with minor modifications. Briefly, 0.5 mL of CBD (2.0 mg/mL) was mixed with 50 μL of 1 M sodium bicarbonate and 100 μL of the AlexaFluor^®^ 555 dye (2.0 mg/mL in DMSO). The reaction mixture was incubated at room temperature for 1 h with constant stirring. Unreacted dye was removed by application to a PD-10 desalting column (GE Healthcare, Cincinnati, OH, USA). The fractions with labeled CBDs were collected and stored at 4 °C for future use to visualize binding.

### 2.11. CBD-Binding Assay

Overnight cultures of bacilli were pelleted at 5000 rpm for 10 min at 4 °C, resuspended in sterile PBS, and washed a second time. Cell suspension aliquots (100 μL) were mixed with 10 μL of each labeled CBDs in separate reactions, and incubated on ice for 10 min. The reaction in absence of fluorescent dye served as a control. After incubation, labeled bacterial cells were pelleted and washed with ice-cold PBS and diluted to 100 μL again. An aliquot (~1 μL) of this mixture was applied to a glass slide, sealed with a glass coverslip, visualized with an Eclipse 80i epifluorescent microscope (Nikon, Tokyo, Japan), and NIS-Elements software (version number 3.22.15, Nikon) was used for image analysis.

### 2.12. Structural Modeling of BACILLUS Bacteriophage Endolysin EADs

The amino acid sequences of PlyP56, PlyN74, and PlyTB40 were submitted to the HHPred server [29] to identify appropriate homology modeling templates of known structures. The phylogenetically closest structurally characterized homolog in the RCSB Protein Data Bank (PDB) [30] was identified and selected from the resulting HHPred hit list for each endolysin EAD based on maximal percent identity. For PlyP56, the l-alanoyl-d-glutamate peptidase from *Listeria monocytogenes* bacteriophage A500, known as Ply500 [22], was selected (PDB ID: 2VO9; 1.8 Å resolution) with 70% identity (E-value = 2 × 10^−75^). For PlyN74, the *N*-acetylmuramoyl-l-alanine amidase from *Bacillus anthracis* λ prophage Ba02, known as PlyL [23], was selected (PDB ID: 1YB0; 1.86 Å resolution) with 53% identity (E-value = 7 × 10^−49^). For PlyTB40, another *N*-acetylmuramoyl-l-alanine amidase with a different fold was selected (PDB ID: 1XOV; 1.8 Å resolution) from *Listeria monocytogenes* bacteriophage PSA, known as PlyPSA [21], with 37% identity (E-value = 3 × 10^−25^). The template and target amino acid sequences for each EAD were subsequently aligned with Clustal X 2.1 [31] using the default parameters. From each alignment (see Figures S1–S3), a percent identity (%I = number of identical alignment positions/total number of alignment positions) and percent similarity (%S = [number of identical alignment positions + number of ‘strong similarity’ alignment positions]/total number of alignment positions) was calculated (PlyN74–1YB0: %I = 51.3, %S = 64.1; PlyP56–2VO9: %I = 70.1, %S = 81.6; PlyTB40–1XOV: %I = 36.3, %S = 53.2). Gaps (i.e., insertions and deletions) were included in the total number of alignment positions. Using the sequence alignments from Clustal X and the template structures from the PDB, the automodel function of MODELLER 9.16 [32,33] was used to generate a population of 100 homology models for each EAD. The model with the lowest Discrete Optimized Protein Energy (DOPE) [34] score from each population was selected for further analysis (PlyN74: DOPE = −16,088; PlyP56: DOPE = −15,027; PlyTB40: DOPE = −16,997). The selected EAD models were postprocessed and visualized with SYBYL-X 2.1.1 (Certara USA, Inc., Princeton, NJ, USA). The models were subjected to a short energy-minimization (Tripos Force Field, Gasteiger−Hückel charges, distance-dependent dielectric constant = 4.0 D/Å, termination criteria: energy gradient cutoff = 0.05 kcal (mol × Å)^−1^ or 200 iterations) followed by generation of Connolly surfaces, onto which the electrostatic potential was mapped. The stereochemical quality of the final models and their corresponding PDB templates were assessed using PROCHECK [35]. In each of the generated endolysin EAD models, >90% of the residues were located in the most favored regions, indicating good quality models.

## 3. Results

### 3.1. Phylogenetic Analysis

**Figure 1:**
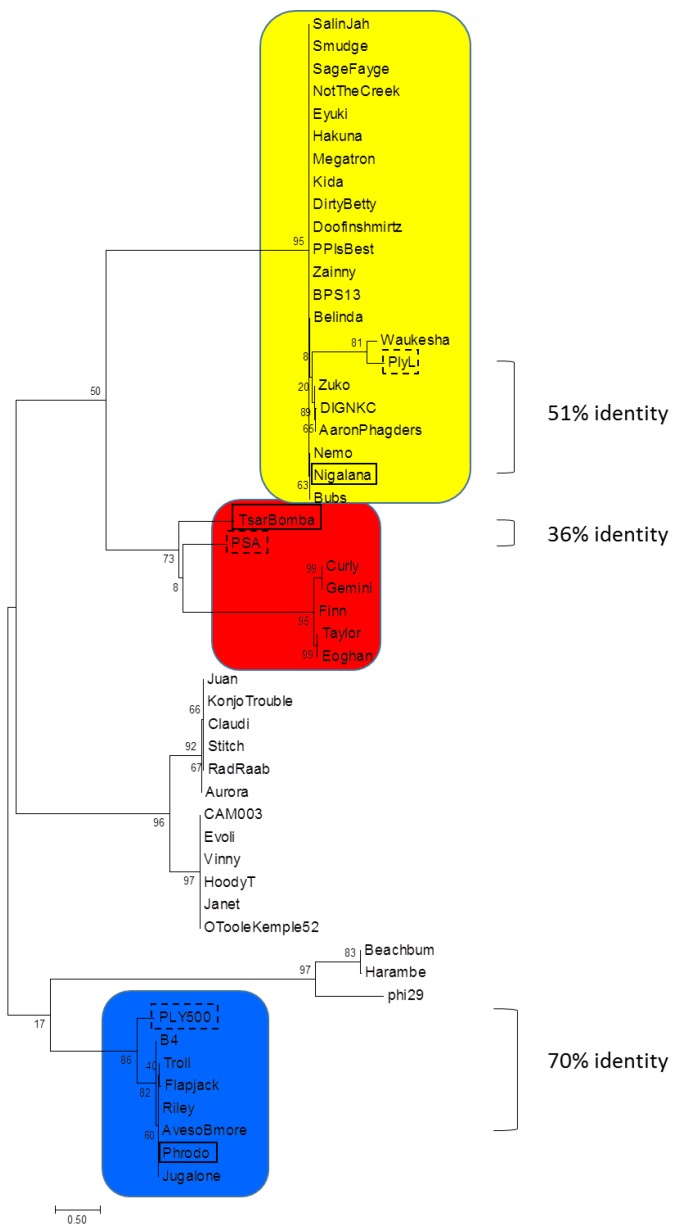
Molecular phylogenetic analysis of bacteriophage endolysin EADs. Sequences were obtained from Bacillus Phage Database (bacillus.phagesdb.org) that have also been deposited into GenBank and compared to six published phage endolysin sequences. Endolysins are represented by their phage names. The evolutionary history was inferred by using the Maximum Likelihood method. The tree is drawn to scale, with branch lengths measured in the number of substitutions per site. Endolysins tested in this manuscript are boxed in black and structurally characterized homologs are boxed in a dashed line. The scale bar represents 0.5 substitutions per amino acid site. Color coding corresponds to EAD domains in Figure 2.

The 46 bacteriophages used in this study were originally isolated, sequenced, and annotated by undergraduate students under the SEA-PHAGES initiative [36] and deposited in the Bacillus Phages Database (bacillus.phagesdb.org). All ORFs were analyzed for genes encoding putative endolysins. Sequences for six biochemically or structurally characterized homologs (LysB4, Ply500, PlyL, PlyPSA, LysBPS13, and phi29) were also included in our analysis [21,22,23,24,25,26]. The 52 enzymes were grouped into nine separate phylogenetic families based on identities and architectural arrangement of the EAD and CBD domains (Table 1). Phylogenetic analysis of the EADs alone indicated four different enzymatic clades (Figure 1). The endolysins from bacteriophages Phrodo, Nigalana, and TsarBomba, called PlyP56, PlyN74, and PlyTB40, respectively, were chosen for expression and further study because they displayed EADs from separate clades but had similar CBDs (see below).

### 3.2. Endolysin Domain Architecture and Homology

**Figure 2:**
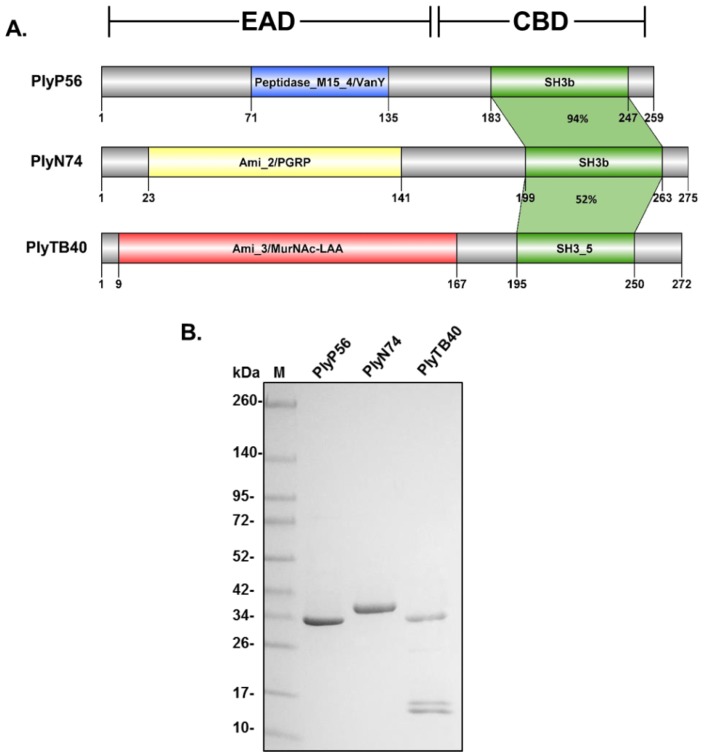
*Bacillus* bacteriophage endolysin structural characterization and protein profile. (**A**) PlyP56, PlyN74, and PlyTB40 contain divergent N-terminal enzymatic active domains (EADs) and conserved C-terminal cell wall binding domains (CBDs). PlyP56 has a Peptidase_M15_4 EAD domain found within the VanY superfamily. PlyN74 has an Amidase_2 EAD domain that is part of the MurNAc-LAA superfamily. PlyTB40 has an Amidase_3 EAD that is also part of the MurNAc-LAA superfamily but lacks homology with the Amidase_2 domain of PlyN74. All three endolysins have similar SH3-family binding domains. Color coding of EADs correspond to Figure 1. (**B**) Purification of *Bacillus* phage endolysins. *E. coli* BL21-(DE3) cells were transformed with a vector encoding recombinant endolysins, grown, and induced with l-arabinose as described under Methods. The recombinant endolysins were purified to homogeneity by nickel affinity chromatography. Protein samples were analyzed for purity by SDS-PAGE with Coomassie blue staining. Lane 1, molecular mass markers as indicated; Lane 2, PlyP56; Lane 3, PlyN74; Lane 4, PlyTB40.

A Pfam database analysis confirmed that PlyP56, PlyN74, and PlyTB40 each contained a singular N-terminal EAD and a C-terminal CBD (Figure 2A). The PlyP56 EAD is predicted to be a member of the Peptidase_M15_4/VanY superfamily (Pfam 13539, Pfam 02557), which is associated with a d-alanyl-d-alanine carboxypeptidase activity. However, such an activity would not readily lead to lysis of the peptidoglycan. Furthermore, the PlyP56 EAD shares significant sequence homology (95% identity) with LysB4 (AFF27501.1), an endolysin from the *B. cereus* bacteriophage B4, which has a confirmed l-alanoyl-d-glutamate endopeptidase activity based on mass spectrometry analysis [26]. The PlyN74 EAD is predicted to belong to the Amidase_2/PGRP superfamily (Pfam 01510) and shares 95% identity to LysBPS13 (AEZ50187.1), a confirmed *N*-acetylmuramoyl-l-alanine amidase from the *B. cereus* bacteriophage BPS13 [24]. These enzymes cleave the amide bond between the glycan component (*N*-acetylmuramic acid) and the peptide component (l-alanine) of the peptidoglycan. Finally, the PlyTB40 EAD is a putative Amidase_3/MurNAc-LAA (pfam 01520). Similar to the Amidase_2 catalytic domain of the PGRP superfamily, the Amidase_3 catalytic domain also possesses an *N*-acetylmuramoyl-l-alanine amidase activity, although this EAD adopts a different fold (Figure S4) [37]. Thus, despite PlyN74 and PlyTB40 containing structurally different catalytic domains (compare Figure 5E,H), both endolysins share a similar enzymatic target—the amide bond between *N*-acetylmuramic acid and l-alanine in the peptidoglycan.

In contrast to the divergent and nonhomologous EADs, all three endolysins are predicted to have a type of src-homology 3 (SH3) domain as their C-terminal CBD (Figure 2A). The CBDs of PlyP56 and PlyN74 have SH3 bacterial domains, known as SH3b domains (smart 00287), which share 94% identity. The PlyTB40 CBD has a very similar SH3_5 domain (pfam 08460) that shares ~52% identity with the SH3b domains of PlyP56 and PlyN74. Notably, SH3b and SH3_5 domains are commonly found CBDs in endolysins derived from bacteriophage that infect Gram-positive bacteria [19], including the *Bacillus*-specific endolysins Ply21 [38] and LysB4 [26].

### 3.3. Purification and Biochemical Characterization

All three endolysins and their corresponding CBDs were expressed as soluble proteins in a pBAD24 expression vector and purified to homogeneity by nickel affinity chromatography via C-terminal 6xHis tags. The size of purified PlyP56, PlyN74, and PlyTB40 bands on SDS-PAGE corresponded to 28.5 kDa, 31.4 kDa, and 30.0 kDa, respectively (Figure 2B). Notably, the PlyTB40 purified protein fraction resulted in a full-length ~30 kDa protein and one or two smaller bands in the ~10–15 kDa range on SDS-PAGE. It should be noted that some clostridial and enterococcal endolysins use alternate translation start sites that generate an additional CBD resulting in the formation of heterodimer enzymes, which would explain the presence of protein bands that correspond to the full-length endolysin and that of a CBD [39,40]. However, we did not detect consensus Shine–Dalgarno sequences or in-frame start codons in the region corresponding to the beginning of the PlyTB40 CBD. Thus, we believe the smaller fragment(s) represent a degradation event despite our use of protease inhibitors during purification.

### 3.4. Activity and Biochemical Characterization of Endolysins

**Figure 3:**
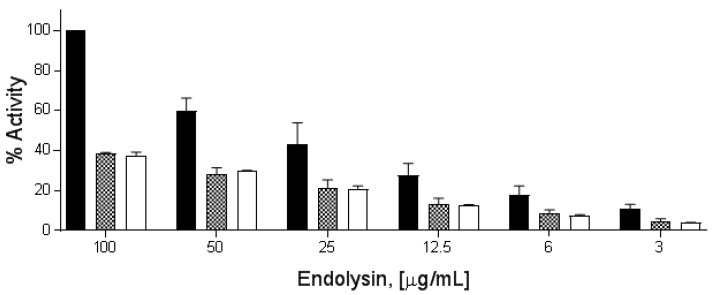
PlyP56, PlyN74, and PlyTB40 lytic activity comparison. Stationary phase *B. cereus* ATCC 4342 cells at final OD_600_ of 1.0 were treated with endolysin doses from 100 μg/mL to 3 μg/mL over 20 min. PlyP56 (black bars), PlyN74 (checker bars), and PlyTB40 (white bars) are indicated. The cell lysis was assayed by turbidity reduction as described in Methods. The % lytic activities were normalized to 100% activity of PlyP56 (black bars) at 100 μg/mL. Experiments were run in triplicates on three independent days. The error bars represent standard deviation.

All three endolysins exhibited a dose–response curve from 100 to 3 μg/mL when tested via the turbidity reduction assay against overnight cultures of *B. cereus* ATCC 4342, with PlyP56 being at least twice as active as PlyN74 and PlyTB40 at all tested concentrations (Figure 3). PlyP56-induced lysis of the bacterial peptidoglycan caused a decrease in OD from 1.0 to 0.4 (60% decrease) within the first 4 min of the turbidity assay at the highest tested dose (100 μg/mL), whereas equimolar concentrations of PlyN74 and PlyTB40 required 10–15 min to achieve the same degree of lysis.

**Figure 4:**
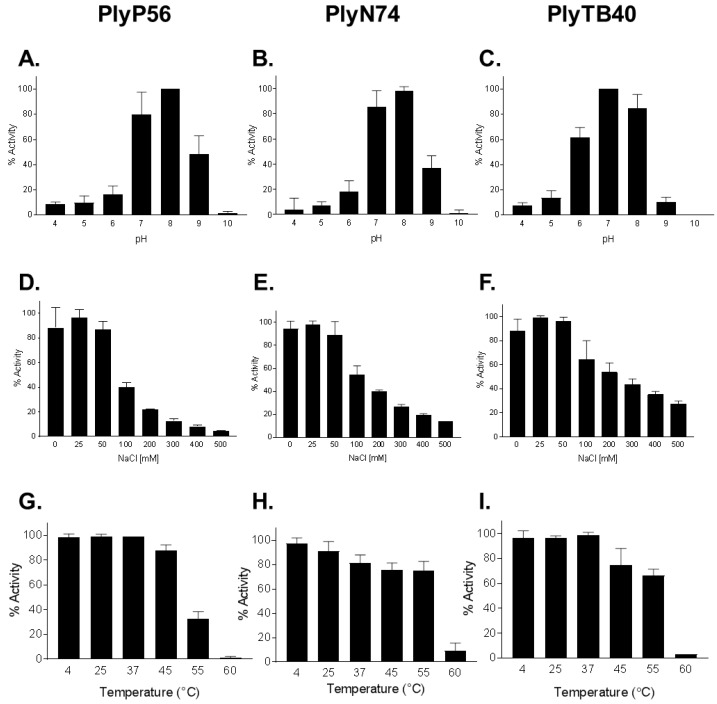
Biochemical characterization of optimal conditions for *Bacillus* bacteriophage endolysins activity. The effects of pH (**A**–**C**), NaCl dependence (**D**–**F**), and temperature stability (**G**–**I**) was evaluated for each of the three endolysins. PlyP56 (**A**,**D**,**G**), PlyN74 (**B**,**E**,**H**), and PlyTB40 (**C**,**F**,**I**) were assayed for lytic activity, each at 50 μg/mL, and tested separately via turbidity reduction assay against stationary phase *B. cereus* ATCC 4342 cells for 20 min. The temperature effect on lytic activity was tested after endolysins were preincubated at indicated temperatures for 30 min and subsequently recovered on ice for 5 min. Values are presented as a percentage of lytic activity in relation to highest activity observed for each tested parameter. The experiments were run in triplicates on three independent days. Error bars indicate standard deviations.

Based on numerous studies, the enzymatic effectiveness of endolysins can often be affected by salt concentration, pH, and temperature [14,19,41,42]. To determine the optimum conditions for PlyP56, PlyN74, and PlyTB40, the lytic activity of these enzymes was surveyed over a broad range of pH (3–11), NaCl concentrations (0–500 mM), and exposure to different temperatures (4–60 °C). In general, all endolysins displayed similar biochemical/biophysical profiles despite possessing different EADs (Figure 4). All three endolysins displayed high lytic activity (90%–100%) at pH 7 and 8, but activity rapidly dropped off outside of this range for PlyP56 and PlyN74 (Figure 4A,B). In contrast, PlyTB40 retained >60% activity at pH 6 and ~40% activity at pH 5 (Figure 4C). These findings suggest a narrower pH range than found in other *Bacillus*-specific endolysins, but nonetheless, they are consistent with a skew toward neutral to basic pH optimums. For instance, PlyPH, a bacteriolytic enzyme identified within the genome of *B. anthracis*, exhibits a relatively broad optimum from pH 5 to 9 [43], whereas LysB4, a PlyP56 homolog, has optimal lytic activity between pH 8.0 and pH 10.5 [26]. LysBPS13, a *B. cereus*-specific endolysin and a homolog of PlyN74, exhibits similar low tolerance to acidic pH below 6.0 [24]. In our experiments, pH extremes not only reduced enzymatic activity of the surveyed endolysins, but it also caused a precipitation of endolysins at the acidic pHs. Taken together, our findings suggest that *Bacillus* species-specific endolysins can sustain their enzymatic activity at a broad pH range but prefer physiological and slightly basic conditions.

The influence of NaCl on enzymatic activity was also studied at pH 7.4, where all the enzymes displayed maximum activity. It was reported that salt concentrations can significantly enhance enzymatic activity of many endolysins [41]. However, NaCl concentrations up to 100 mM had little effect (<10% deviation) on the lytic activity of PlyP56, PlyN74, and PlyTB40 (Figure 4D–F). A similar effect was observed for staphylococcal endolysin, PlyGRCS [44], which displayed full activity up to 500 mM NaCl. On the contrary, NaCl concentrations equal to or above 100 mM significantly inhibited enzymatic activity of all three enzymes, with PlyP56 being the most sensitive, losing half of its activity at just 100 mM NaCl. PlyTB40 was the least sensitive to NaCl of the three enzymes, but still lost half of its lytic activity at 300 mM.

The thermal stability of each endolysin was determined by incubation at temperatures ranging from 4 to 60 °C, recovering on ice, and measuring residual activity by the turbidity reduction assay. It was determined that all endolysins were enzymatically active over a temperature range from 4 to 45 °C, with minor deviations in activity (±15% of maximum) (Figure 4G–I). At 55 °C, PlyN74 and PlyTB40 maintained >80% of maximum activity whereas PlyP56 displayed <40% of maximum activity. By 60 °C, all three endolysins had <10% lytic activity remaining. In general, the thermal stability profile of PlyP56, PlyN74, and PlyTB40 was found to be consistent with other *Bacillus* endolysins. For instance, LysBPS13 and BtCS33 were inactivated after a 30 min incubation at 60 °C in the absence of thermoprotective agents [42].

### 3.5. Structural Modeling of Bacillus Bacteriophage Endolysin EADs

**Figure 5:**
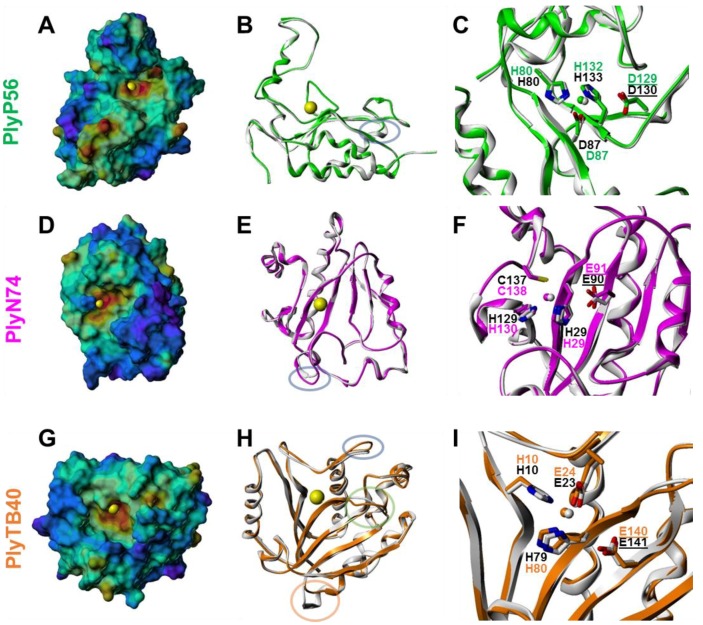
Homology models of PlyP56 (**A**–**C**), PlyN74 (**D**–**F**), and PlyTB40 (**G**–**I**) EADs. (**A**,**D**,**G**) Connolly surface representations color-coded by electrostatic potential (blue = most positive; red = most negative). A yellow sphere represents the Zn^2+^ ion. (**B**,**E**,**H**) Ribbon representations of the homology modeling template (PlyP56: PDB ID = 2VO9; PlyN74: PDB ID = 1YB0; PlyTB40: PDB ID = 1XOV; white) and target (PlyP56, green; PlyN74, magenta; PlyTB40, orange) EADs illustrating the putative protein fold conservation. Colored ovals represent sequence insertions or deletions; see Figures S1–S3. (**C**,**F**,**I**) Catalytic active site amino acid residues. Residue label colors represent template (black) and target (PlyP56, green; PlyN74, magenta; PlyTB40, orange) EADs. An underline indicates the catalytic base/acid. Small spheres represent the Zn^2+^ ion.

We used homology modeling techniques (see Methods) to generate plausible three-dimensional models of the PlyP56, PlyN74, and PlyTB40 EADs (Figure 5). Each model fit its template well, with complete conservation of catalytic residues, moderate to high conservation of noncatalytic amino acids, and only a few small insertions or deletions in loop regions. The differences in amino acid composition on the surfaces of closely related EAD family members are responsible for differences in their shape and electrostatic nature (see e.g., Figures S1–S3). These factors in turn contribute to differences in the functional protein–protein interactions and catalytic specificity exhibited by members within and between the various endolysin fold families [37,45,46]. Taken together, the 3-D structural modeling results support the functional prediction of *N*-acetylmuramoyl-l-alanine amidase activity for the PlyN74 and PlyTB40 endolysins, while clarifying PlyP56 as an l-alanoyl-d-glutamate peptidase.

### 3.6. Effect of Divalent Metal Ions

Based on 3-D modeling, all three endolysin EADs are predicted to have a characteristic monometallic metallopeptidase-like catalytic active site in which a Zn^2+^ ion is tetrahedrally coordinated by three conserved amino acid residues and a water molecule, and that also contains an adjacent catalytic base/acid, usually Asp or Glu [47]. For PlyP56, the Zn^2+^-coordinating residues are His80, Asp87, and His132, and the catalytic base/acid is Asp129 (Figure 5C). For PlyN74, the Zn^2+^-coordinating residues are His29, His130, and Cys138, and the catalytic base/acid is Glu91 (Figure 5F). For PlyTB40, the Zn^2+^-coordinating residues are His10, Glu24, and His80, and the catalytic base/acid is Glu140 (Figure 5I). Significantly, Ply500 contains a conserved metal binding sequence (SxHxxGxAxD) and its crystal structure revealed an ion in the active site [22]. Sequence alignment also detected this motif in PlyP56 (Figure S1A). Although not necessarily associated with a specific sequence motif, the metal binding site in Amidase_2 and Amidase_3 *N*-acetylmuramoyl-l-alanine amidases has been structurally characterized using X-ray crystallography, and strictly conserved metal-coordinating residues have been identified [37]. The sequence similarity of PlyN74 and PlyTB40 to Amidase_2 and Amidase_3 *N*-acetylmuramoyl-l-alanine amidases and the high quality of the resulting MODELLER-generated models provide strong evidence that these metal binding sites are present in these endolysin EADs as well.

**Figure 6:**
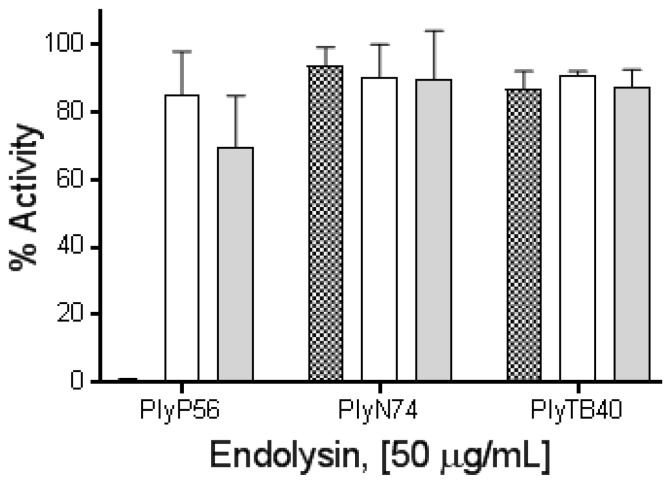
Metal binding properties of PlyP56, PlyN74, and PlyTB40. The influence of divalent cations on PlyP56, PlyN74, and PlyTB40 lytic activity against stationary phase *B. cereus* ATCC 4342 was assayed via turbidity reduction assay. Mean values from three independent experiments run in triplicate are represented as the percentage residual lytic activity relative to untreated endolysins. Endolysins treated with EDTA (checker bars), and subsequently recovered via dialysis with additions of divalent ions, Mg^2+^ (white bars), Ca^2+^ (grey bars) are shown.

To further elucidate these findings, PlyP56, PlyN74, and PlyTB40 were dialyzed overnight in buffer supplemented with 5 mM EDTA to remove residual metal ions. Interestingly, EDTA treatment completely ablated enzymatic activity of PlyP56 but had no effect on the activities of PlyN74 or PlyTB40 (Figure 6). Further, EDTA-treated proteins were dialyzed overnight in TBS supplemented with an excess of metal relative to the EDTA (i.e., 6 mM Mg^2+^ or 6 mM Ca^2+^) to restore cations in these enzymes. Lytic activity of PlyP56 was restored to 80% of the pre-EDTA levels by Mg^2+^ ions and to 70% by Ca^2+^ ions (Figure 6). Our EDTA results are consistent with those found for LysB4, an EAD sequence homolog of PlyP56, which had activity restored to EDTA-treated samples by the addition of Mg^2+^ or Ca^2+^ ions [26]. This confirms that PlyP56 requires divalent metal ions for its enzymatic activity.

In contrast to the PlyP56 results, EDTA treatment had no effect on the enzymatic activity of PlyN74 despite an ion being present in the active site of the crystal structure for PlyL, a homolog of the PlyN74 EAD [23]. However, EDTA-treated LysBPS13, another PlyN74 homolog, was similarly not dependent on the presence of metal ions for activity [24]. Finally, we found that PlyTB40 was also not affected by EDTA, even though a Zn^2+^ ion was identified in the crystal structure of an homologous PlyPSA [21].

### 3.7. Host Specificity

To determine the host range of PlyP56, PlyN74, and PlyTB40, lytic activity was tested via turbidity assay on a variety of *B. cereus* strains and other *Bacillaceae* (Table 2). Similar to the dose–response curves for *B. cereus* ATCC 4342 (Figure 2), PlyP56 was more effective in lysing *B. cereus sensu lato* group species than PlyN74 or PlyTB40, but all three enzymes displayed strong activity, defined as >20% lysis in the 20 min assay period, against all *sensu lato* members tested (i.e., four *B. cereus* strains and one *B. thuringiensis* strain). In addition, all three enzymes showed strong activity against *Bacillus pumilus* strain BJ0050, PlyP56 and PlyN74 both showed strong activity against *Bacillus megaterium* and *Bacillus amyloliquefaciens*, PlyN74 showed strong activity against *Bacillus licheniformis*, and PlyP56 showed strong activity against *Bacillus circulans* and *Lysinbacillus sphaericus*. Weak, but measurable, activity was noted for all three enzymes against *Bacillus coagulans*, *Bacillus subtilis*, and *Paenibacillus polymyxa*.

*B. cereus* ATCC 4342 is a transition state strain that is phylogenetically located between *B. cereus* and *B. anthracis* [48], and as such, we expected our enzymes to be equally effective in cell lysis of *B. anthracis.* However, using the same set of parameters employed for assays in Table 2, we did not observe lytic activity in a turbidity reduction assay against biosafety level 2 *B. anthracis* strains (34F2 Sterne, Ames35, and UM23) or the biosafety level 3 *B. anthracis* Ames strain. However, lytic activity measured via a plate lysis assay, which tends to be more sensitive given longer incubation times, did reveal significant lysis of *B. anthracis* Ames35 bacilli by PlyP56 and PlyN74 with lesser activity against the *B. anthracis* UM23 strain (Table 3). PlyTB40, on the other hand, had very low activity against these strains. Collectively, our findings suggest PlyP56, PlyN74, and PlyTB40 have targeted lytic activity against the *B. cereus sensu lato* group and a few closely related species.

### 3.8. Cell Wall Binding

**Figure 7:**
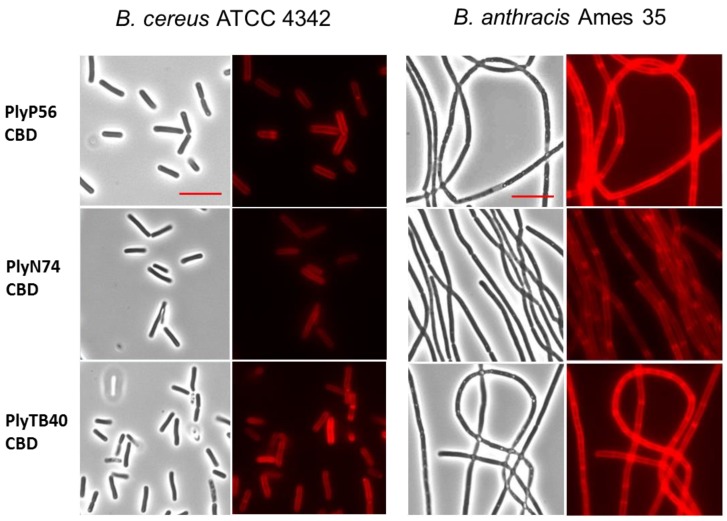
Binding of AlexaFluor-labeled CBDs to a cell wall of bacilli. Decoration of *B. cereus* ATCC 4342 (two left columns) and *B. anthracis* Ames 35 (two right columns) by fluorescently tagged CBDs of PlyP56, PlyN74, and PlyTB40. 1000× bright-field images (columns 1 and 3) are shown with their corresponding fluorescent images (columns 2 and 4). The AlexaFluor-labeled CBDs recognize and bind to an evenly distributed ligand on the surface of *B. cereus* and *B. anthracis*. Red scale bar = 5 μm.

As with many endolysins, the SH3b and SH3_5 domains present in PlyP56, PlyN74, and PlyTB40 are thought to function as their CBDs. To test this hypothesis, we chemically crosslinked the CBDs of these enzymes with AlexaFluor555, purified the crosslinked CBDs, and assessed their binding properties by fluorescent microscopy. All three CBDs bound tightly to the peptidoglycan of *B. cereus* ATCC strains 4342, 14579, 11778, and 13061. Binding to strain 4342 is shown in Figure 7 (two left columns), but similar binding was observed for all *B. cereus* strains. Additionally, all three CBDs bound tightly to the peptidoglycan of the *B. anthracis* strains UM23 and Ames35, with the later depicted in Figure 7 (two right columns). In contrast, none of the CBDs bound non-*sensu lato* strains of *Bacillus*, including *B. licheniformis* ATCC 14580 and *B. coagulans* ATCC 7050. Likewise, the CBDs did not bind other representative Gram-positive bacteria, such as *Streptococcus pyogenes* D471.

## 4. Discussion

Bacteriophage-encoded endolysins are of great interest for their potential as antimicrobial agents useful for controlling bacterial infections and preventing biofilm formation [20,49,50]. They can also be used for unwanted food contamination by opportunistic or pathogenic bacteria [51]. In this paper, we have identified and characterized three *B. cereus* specific endolysins, PlyP56, PlyN74, and PlyTB40, which share basic structural properties of an N-terminal conserved EAD and a C-terminal CBD.

PlyP56 is predicted to have an l-alanoyl-d-glutamate peptidase activity derived from the Peptidase_M15_4/VanY superfamily EAD domain. Sequence analysis identified a conserved (SxHxxGxAxD) motif within the PlyP56 EAD that plays an active role in harboring a metal ion, as first described for VanX of *Enterococcus faecium* [52] and supported by our modeling studies with the Ply500 structural homolog (Figure 5A). As predicted, the PlyP56 lytic activity was abolished by EDTA treatment, which was subsequently restored by addition of excess Mg^2+^ or Ca^2+^ ions. The PlyN74 Amidase_2/PGRP superfamily EAD and the PlyTB40 Amidase_3/MurNAc-LAA superfamily EAD are not homologous and arise from different phylogenetic clades (Table 1), but they nonetheless are predicted to possess identical *N*-acetylmuramoyl-l-alanine amidase activities, suggesting convergent evolution of these superfamily domains. Our modeling to structural homologs for both of these EADs suggested a metal binding pocket with active site residues similar to those of the PlyP56 EAD. However, we were unable to inhibit lytic activity of these two endolysins by EDTA treatment. This discovery suggests that enzymatic activity of both endolysins is independent from metal ions, as proposed by Park et al. [24] for the PGRP superfamily. Alternatively, it is possible that the affinity of the metal ion to the coordinating residues was too strong, and/or the accessibility of the metal binding pocket was too limited, to be susceptible to chelation by EDTA.

PlyP56, PlyN74, and PlyTB40 had very similar biochemical, biophysical, and binding/host range characteristics. The similar binding patterns of these endolysins were anticipated since they all had similar SH3-family CBDs and were originally selected due to high lytic activity on the same *B. cereus* ATCC 4342 indicator strain. However, all three endolysins have distinct EADs, so it is somewhat surprising that their pH, NaCl sensitivity, and temperature stability profiles overlap to a large degree. Given that PlyP56 displays twice the activity of PlyN74 and PlyTB40, it is inviting to speculate that the Peptidase_M15_4 EAD of PlyP56 is more efficient than the Amidase_2 or Amidase_3 EADs of PlyN74 and PlyTB40, respectively. This is further supported by the near-identical CBDs shared by PlyP56 and PlyN74, suggesting these enzymes are only differentiated by their EADs. However, the differences in charge of the EADs cannot be discounted as contributing to the observed differences in activity. A number of studies have reported correlation between the charge of an EAD and its enzymatic activity [53]. Truncated, positively charged EADs of PlyL and CD27L were reported to have higher bactericidal activity and broader host spectrum than their wild-type precursors [54,55]. Remarkably, at neutral pH, the PlyP56 EAD and linker sequence (residues 1–173) would have a predicted net positive charge (pI = 8.55), the PlyN74 EAD/linker (residues 1–189) would have a neutral charge (pI = 7.02), and the PlyTB40 EAD/linker (residues 1–190) would have a slight negative charge (pI = 6.28). It is possible that differences in charge of the EADs, specifically positively charged EADs, may enhance binding properties of the CBDs to negatively charged wall teichoic acids on the bacterial surface and contribute to observed lytic activity, although additional experimentation will be needed to determine if that is the case with the enzymes described here.

It is noteworthy that PlyP56, PlyN74, and PlyTB40 had higher activity against *B. cereus* ATCC 4342 than they did against any other bacilli. Significantly, *B. cereus* ATCC 4342 is a known transition state strain between *B. cereus* and *B. anthracis*, and it is the only *B. cereus* strain lysed by the PlyG endolysin, which lyses all *B. anthracis* strains. Therefore, we expected that the newly discovered endolysins presented here would also be active against *B. anthracis* strains. Although we observed strong binding to *B. anthracis* via all three endolysin CBDs (Figure 7, right two columns) as well as activity against *B. anthracis* in a spot lysis assay for the full-length proteins (Table 3), the overall lytic activity of PlyP56, PlyN74, and PlyTB40 against *B. anthracis* species is considered quite weak since we never observed activity in a liquid turbidity reduction assay. At present, we do know if this diminished activity is related to assay conditions, the strains we chose for study, differences in the peptidoglycan between members of the *sensu lato* group, or the SH3-based CBDs present in our enzymes. Additionally, PlyG is not homologous to any of our endolysins, and PlyL, another well-characterized endolysin with high activity against *B. anthracis* [23], shares only 53% identity with the EAD of PlyTB40. Moreover, the absence of homology between the CBDs of our enzymes and that of characterized *B. anthracis* endolysins suggests they do not share common epitopes. Further work is needed to fully elucidate the interactions between *Bacillus*-specific endolysin EADs and their CBDs.

This study contributes three new *Bacillus*-specific endolysins to the growing toolbox of EADs and CBDs that can be evolved, modified, or shuffled through chimeragenesis to create new enzymes. Despite similar biochemical profiles acquired for PlyP56, PlyN74, and PlyTB40, PlyP56 is the most enzymatically active and has a broader host range than PlyN74 and PlyTB40, which makes it a good lead candidate for further antimicrobial development and bioengineering studies.

## Figures and Tables

**Table 1:** Phylogenetic analysis of 46 *Bacillus* bacteriophage endolysins.

Family	EAD	CBD	Examples
I.	G25 muramidase	Amidase02_C	Vinny ORF63
II.	G25 PlyB-like	Amidase02_C	Stitch ORF31
III.	MurNAc-LAA	2X LysM	Taylor ORF31
IV.	MurNAc-LAA	SH3	TsarBomba ORF40 (PlyTB40)
V.	VanY	2X PG_binding_1	SPO1 ORF107
VI.	Peptidase M15_4/VanY	SH3	Phrodo ORF56 (PlyP56)
VII.	GH24 muramidase	SH3	Beachbum ORF23
VIII.	PGRP	Amidase02_C	Waukesha ORF68
IX.	PGRP	SH3	Nigalana ORF74 (PlyN74)

**Table 2:** Relative lytic activity of *Bacillus* bacteriophage endolysins.

Species	Strain ^1^	Bacteriophage Endolysins ^2^		
		PlyP56	PlyN74	PlyTB40
***B. cereus***	**ATCC 4342**	**84.9 ± 6.0**	**69.2 ± 9.8**	**71.9 ± 12.9**
***B. cereus***	**ATCC 14579**	**73.4 ± 1.3**	**59.7 ± 7.2**	**40.9 ± 20.5**
***B. cereus***	**ATCC 11778**	**79.6 ± 4.4**	**60.6 ± 4.4**	**58.2 ± 13.2**
***B. cereus***	**ATCC 13061**	**45.8 ± 2.4**	**37.9 ± 3.8**	**24.0 ± 8.1**
***B. thuringiensis***	**ATCC 10792**	**38.7 ± 5.2**	**35.8 ± 9.1**	**25.6 ± 6.6**
*B. amyloliquefaciens*	ATCC 23842	36.5 ± 20.5	23.9 ± 13.8	6.2 ± 2.4
*B. circulans*	ATCC 4513	53.2 ± 3.2	17.2 ± 5.1	5.5 ± 5.1
*B. coagulans*	ATCC 7050	8.0 ± 1.7	5.7 ± 7.9	4.6 ± 4.5
*B. licheniformis*	ATCC 14580	4.6 ± 6.4	30.2 ± 3.6	9.6 ± 3.7
*B. megaterium*	ATCC 14581	83.9 ± 12.3	30.2 ± 15.5	9.6 ± 1.6
*B. pumilus*	BJ0050	58.8 ± 15.2	46.1 ± 11.1	32.6 ± 14.4
*B. pumilus*	ATCC 700814	16.7 ± 18.4	10.9 ± 12.5	2.6 ± 4.3
*B. subtilis*	ATCC 6051	3.5 ± 1.9	1.4 ± 0.5	0.1 ± 0.2
*B. subtilis*	ATCC 33608	2.9 ± 2.3	1.6 ± 2.7	0.6 ± 0.8
*Lysinb. Sphaericus*	ATCC 4525	36.9 ± 19.8	18.9 ± 9.4	10.8 ± 3.4
*Paenib. Polymyxa*	ATCC 7070	6.0 ± 4.7	3.7 ± 4.3	3.1 ± 1.5

^1^ See Methods for source of species and strains. Strains in bold belong to the *B. cereus sensu lato* group. ^2^ Activity of endolysins was evaluated via turbidity reduction assay. Values reported are the percent decrease in absorbance (OD_600_) of cells treated with endolysins normalized to values of untreated cells after 20 min incubation with 100 μg/mL of each endolysin. The starting absorbance of mid-log cells was adjusted to an OD_600_ of 1.0. Values represent mean values from three independent experiments, run in triplicate.

**Table 3:** Plate lysis.

Species	Strain ^1^	Bacteriophage Endolysins ^2^		
		PlyP56	PlyN74	PlyTB40
*B. cereus*	ATCC 4342	+++	+++	++
*B. anthracis*	Ames 35	++	+++	+
*B. anthracis*	UM23	+	+/−	+/−

^1^ See Methods for source of species and strains. ^2^ Activity of endolysins was evaluated via plate lysis assay. 10 μL of each endolysin containing 10, 1, or 0.1 μg were spotted onto a surface of semisolid agar containing a mid-log bacterial cell suspension. The strength of lysis was defined by the presence of a clearing zone: +/−, for a partial clearing zone at 10 μg; +, for a clearing zone at 10 μg; ++, for a clearing zone at 1 μg; and +++, for a clearing zone at 0.1 μg. PBS was spotted in equal volumes and served as a negative control.

## Supplementary Materials

The following are available online at http://www.mdpi.com/1999-4915/10/5/276/s1, Figure S1: PlyP56 alignment with structural homolog Ply500; Figure S2: PlyN74 alignment with structural homolog PlyL, Figure S3: PlyTB40 alignment with structural homolog PlyPSA; Figure S4: Carboxypeptidase T-type Amidase_3 fold; Table S1: Primers used in this study.
