# Supplementary material for: Discovery and Biochemical Characterization of PlyP56, PlyN74, and PlyTB40—Bacillus Specific Endolysins
Source: Viruses. 2018 May 21;10(5):276. doi: 10.3390/v10050276 (PMC5977269; doi:10.3390/v10050276)
Supplement: Supplementary file 1 [file viruses-10-00276-s001.pdf]

A

```
                                 10         20         30
                         ....|....| ....|....| ....|....|
2vo9_a        1          MALTEAWLIE KANRKLNAGG MYKITSDKTR   30
PlyP56        1          MAMALQTLID KANRKLNISG MRKDVADRTR   30
                         **::   **: ******* .* * * .:*:**

                                 40         50         60
                         ....|....| ....|....| ....|....|
2vo9_a        31         NVIKKMAKEG IYLCVAQGYR STAEQNALYA   60
PlyP56        31         AVITQMHAQG IYICVAQGFR SFAEQDALYA   60
                          **.:*  :* **:*****:* * ***:****

                                 70         80         90
                         ....|....| ....|....| ....|....|
2vo9_a        61         QGRTKPGAIV TNAKGGQSNH NYGVAVDLCL   90
PlyP56        61         QGRTKPGNIV TNARGGQSNH NYGVAVDLCL   90
                         ******* ** ***:****** **********

                                100        110        120
                         ....|....| ....|....| ....|....|
2vo9_a        91         YTNDGKDVIW ESTTSRWKKV VAAMKAEGFK   120
PlyP56        91         YTQDGSDVIW TVEGN-FRKV IAAMKGQGFK   119
                         **:**.****     . ::** :****.:***

                                130        140
                         ....|....| ....|....| ....|..
2vo9_a        121        WGGDWKSFKD YPHFELCDAV SGEKIPA 147
PlyP56        120        WGGDWVSFKD YPHFELYDVV GGQKPPA 146
                         ***** **** ****** *.* .*:* **
```

B **C**

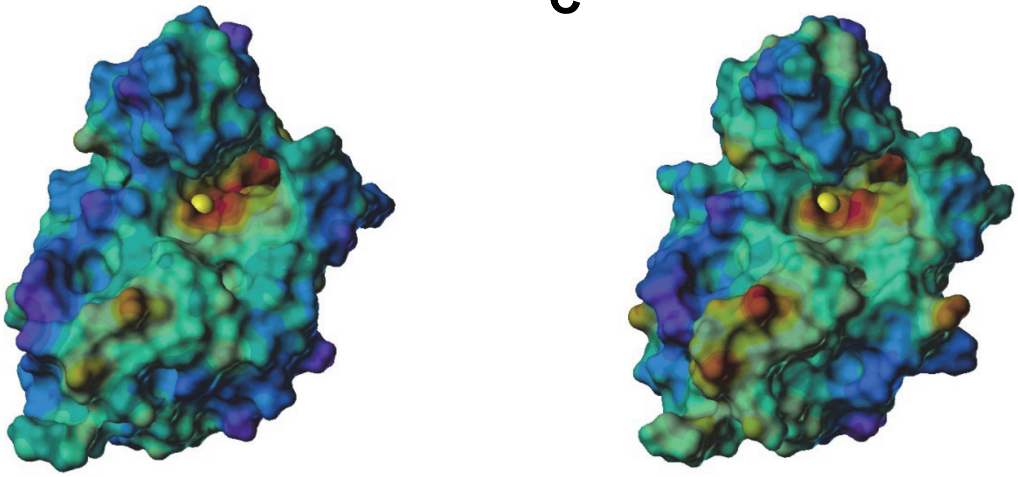

Figure S1. PlyP56 alignment with structural homolog Ply500. (A) Sequence alignment of *L. monocytogenes* phage A500 (Ply500) from PDB entry 2VO9 and PlyP56 EADs (Clustal X alignment symbols: asterisk = identical; colon = strongly similar; period = weakly similar; space = not similar). Overall percent identity (# identical / # total) = 70.1%; percent similarity [(# identical + # strongly similar) / # total] = 81.6%. Arrows indicate the metal-binding residues (red) and the catalytic base/acid (blue). The conserved SxHxxGxAxD zinc-binding motif is highlighted with a red rectangle. Colored ovals represent sequence insertions or deletions; see Fig. 5. **(B–C)** Connolly surfaces color-coded by electrostatic potential (blue = most positive; red = most negative) for the template Ply500 **(B)** and the modeled PlyP56 **(C)** EAD; a yellow sphere represents the Zn^{2+} ion.

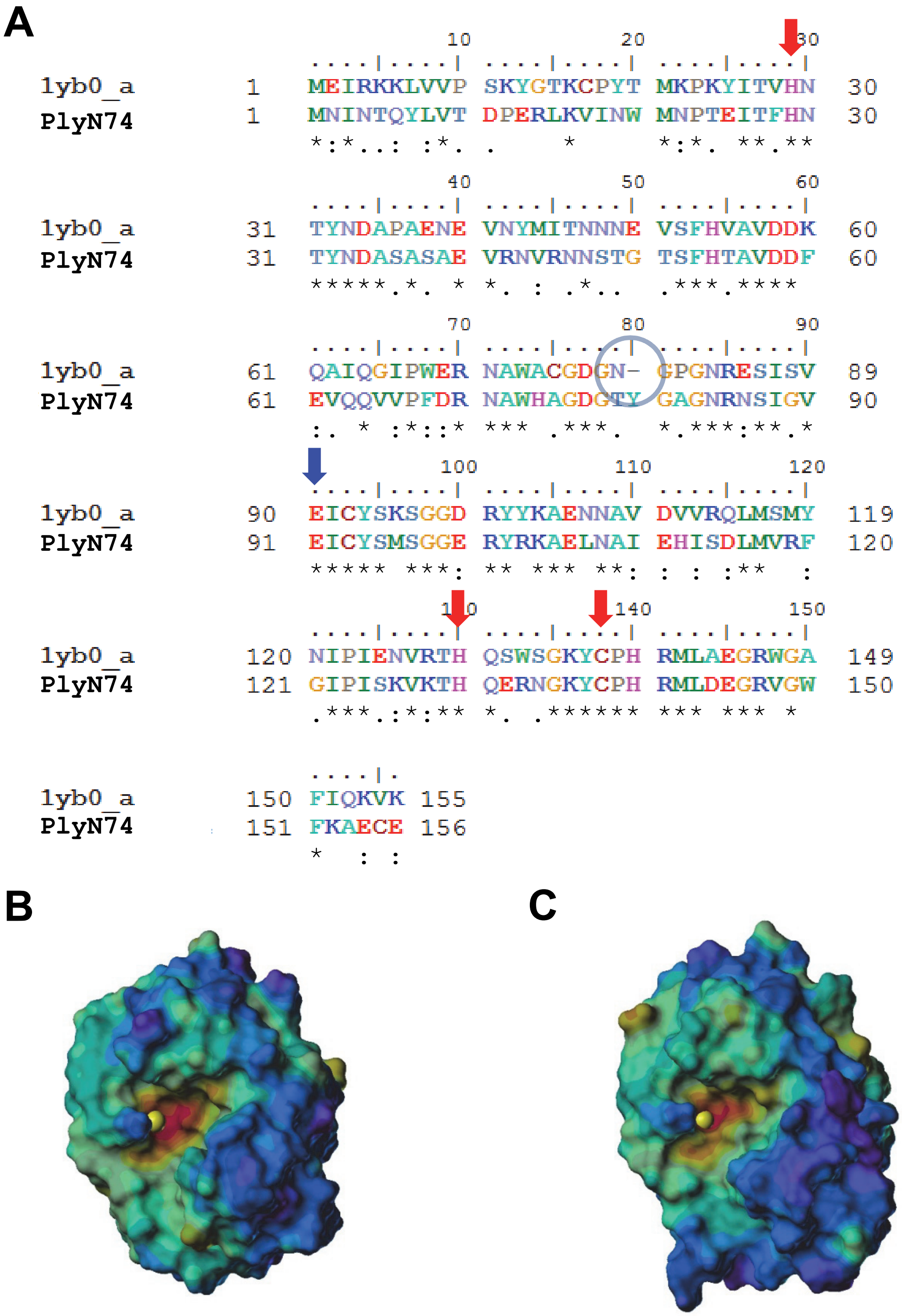

Figure S2. PlyN74 alignment with structural homolog PlyL. (A) Sequence alignment of *B. anthracis* λ prophage Ba02 (PlyL) from PDB entry 1YB0 and PlyN74 EADs (Clustal X alignment symbols: asterisk = identical; colon = strongly similar; period = weakly similar; space = not similar). Overall percent identity (# identical / # total) = 51.3%; percent similarity [(# identical + # strongly similar) / # total] = 64.1%. Arrows indicate the metal-binding residues (red) and the catalytic base/acid (blue). Colored ovals represent sequence insertions or deletions; see Fig. 5. **(B–C)** Connolly surfaces color-coded by electrostatic potential (blue = most positive; red = most negative) for the template PlyL **(B)** and the modeled PlyN74 **(C)** EAD; a yellow sphere represents the Zn^{2+} ion.

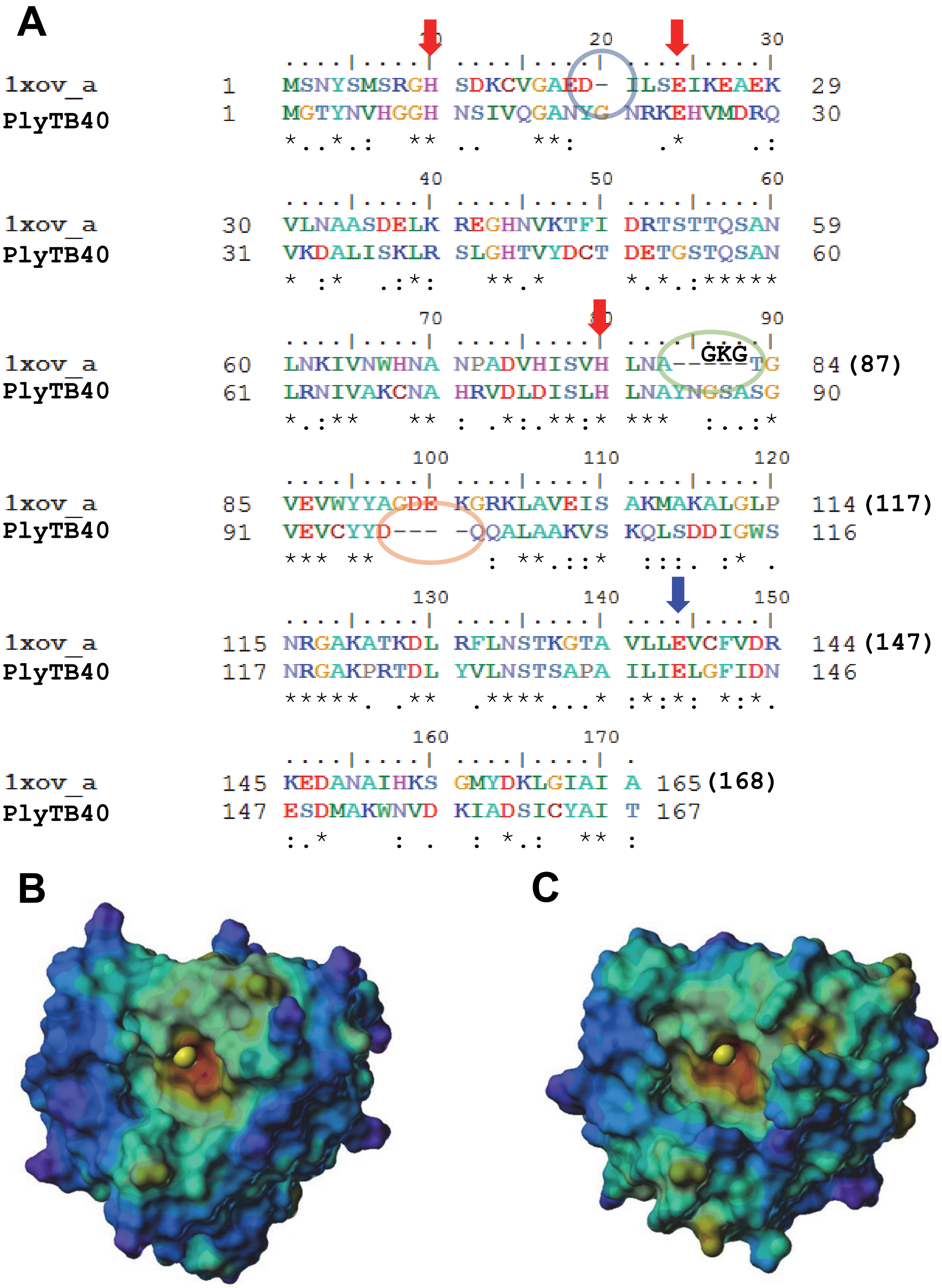

Figure S3. PlyTB40 alignment with structural homolog PlyPSA. (A) Sequence alignment of *L. monocytogenes* phage PSA (PlyPSA) from PDB entry 1XOV and PlyTB40 EADs (Clustal X alignment symbols: asterisk = identical; colon = strongly similar; period = weakly similar; space = not similar). Overall percent identity (# identical / # total) = 36.3%; percent similarity [(# identical + # strongly similar) / # total] = 53.2%. Arrows indicate the metal-binding residues (red) and the catalytic base/acid (blue). Colored ovals represent sequence insertions or deletions; see Fig. 5. **(B–C)** Connolly surfaces color-coded by electrostatic potential (blue = most positive; red = most negative) for the template PlyPSA **(B)** and the modeled PlyTB40 **(C)** EAD; a yellow sphere represents the Zn^{2+} ion.

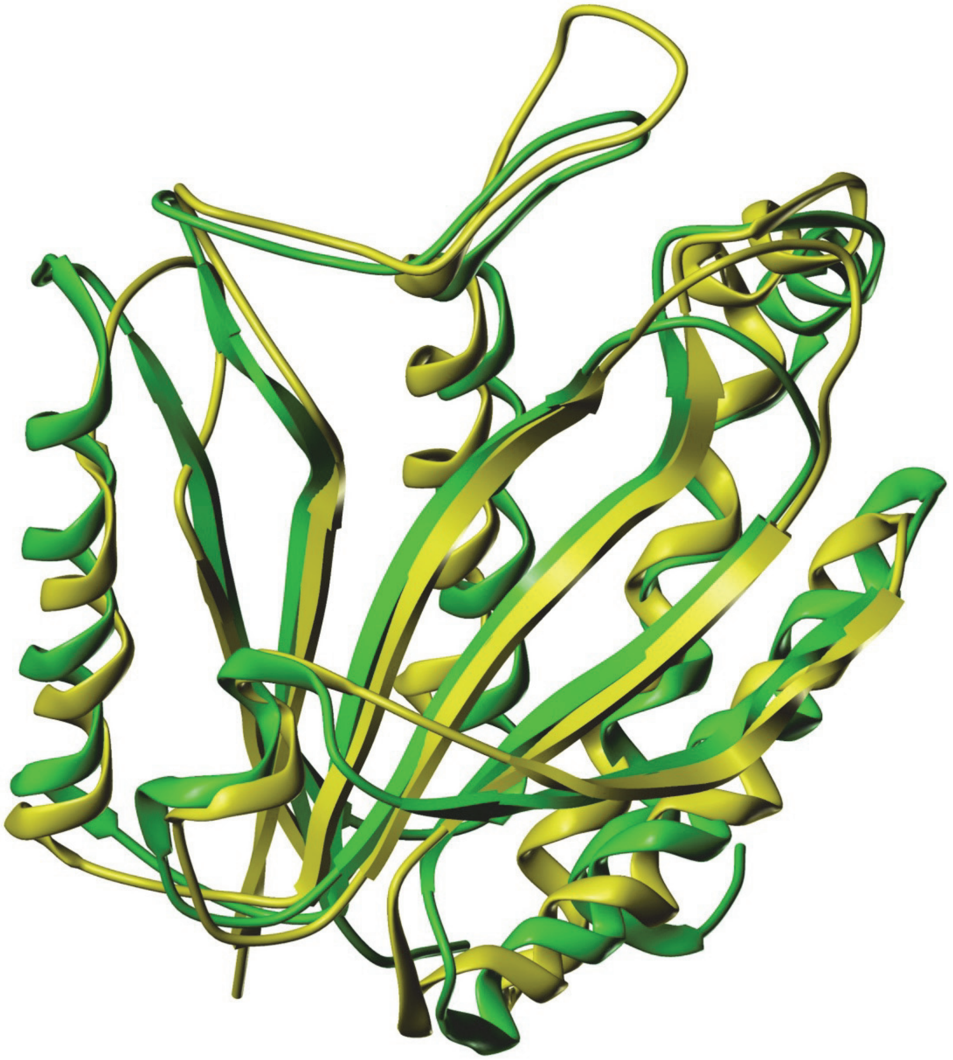

Figure S4. Carboxypeptidase T-type Amidase_3 fold. Ribbon diagrams of the endolysin EADs of *Listeria monocytogenes* bacteriophage PlyPSA (PDB ID: 1XOV; PlyTB40 homology modeling template; green) and *Bacillus polymyxa* var. *colistinus* CwlV (PDB ID: 1JWQ; yellow), each exhibiting the carboxypeptidase T-type Amidase_3 fold.

Supplementary Material

Table S1: Primers used in this study

**Template**	**Primer**	**Sequence (5' > 3')***
pBAD24::*plyP56*	IR29 F'	CGTGAATTCATGCATCATCATCATCATCATGATTATGATAGCAGCTGG
	IR30 R'	CGTTCTAGATTATTTAAACGTGCCCCAATA
pBAD24::*plyN74*	IR33 F'	CGTGAATTCATGCATCATCATCATCATCATGATTATGATAGCAGCTGGTTTACC
	IR34 R'	CGTTCTAGATTATTTAAAGGTGCCCCAATAATTCAC
pBAD24::*plyTB40*	IR37 F'	CGTGAATTCATGCATCATCATCATCATCATTATGATAGCAGCTGGTTTACACCG
	IR38 R'	CGTTCTAGATTACTGAAAGGTGCCCCAATAGCTAAT

* Restriction sites underlined
